# Analysis of the influence of imaging-related uncertainties on cerebral aneurysm deformation quantification using a no-deformation physical flow phantom

**DOI:** 10.1038/s41598-018-29282-0

**Published:** 2018-07-20

**Authors:** Daniel Schetelig, Jan Sedlacik, Jens Fiehler, Andreas Frölich, Tobias Knopp, Thilo Sothmann, Jonathan Waschkewitz, René Werner

**Affiliations:** 1 University Medical Center Hamburg-Eppendorf, Department of Computational Neuroscience, Hamburg, 20246 Germany; 2 University Medical Center Hamburg-Eppendorf, Department of Diagnostic and Interventional Neuroradiology, Hamburg, 20246 Germany; 3 University Medical Center Hamburg-Eppendorf, Section for Biomedical Imaging, Hamburg, 20246 Germany; 4 Hamburg University of Technology, Institute for Biomedical Imaging, Hamburg, 20246 Germany; 5 University Medical Center Hamburg-Eppendorf, Department of Radiotherapy and Radiation Oncology, Hamburg, 20246 Germany

## Abstract

Cardiac-cycle related pulsatile aneurysm motion and deformation is assumed to provide valuable information for assessing cerebral aneurysm rupture risk. Accordingly, numerous studies addressed quantification of cerebral aneurysm wall motion and deformation. Most of them utilized *in vivo* imaging data, but image-based aneurysm deformation quantification is subject to pronounced uncertainties: unknown ground-truth deformation; image resolution in the order of the expected deformation; direct interplay between contrast agent inflow and image intensity. To analyze the impact of the uncertainties on deformation quantification, a multi-imaging modality ground-truth phantom study is performed. A physical flow phantom was designed that allowed simulating pulsatile flow through a variety of modeled cerebral vascular structures. The phantom was imaged using different modalities [MRI, CT, 3D-RA] and mimicking physiologically realistic flow conditions. Resulting image data was analyzed by an established registration-based approach for automated wall motion quantification. The data reveals severe dependency between contrast media inflow-related image intensity changes and the extent of estimated wall deformation. The study illustrates that imaging-related uncertainties affect the accuracy of cerebral aneurysm deformation quantification, suggesting that *in vivo* imaging studies have to be accompanied by ground-truth phantom experiments to foster data interpretation and to prove plausibility of the applied image analysis algorithms.

## Introduction

Cerebral aneurysms are anomalous dilated arteries with a potentially severe complication: rupture^1^. Upon rupture, high moderate-to-severe disability and short-term mortality rates have been reported (35% and 29%^2^); however, the estimated incidence of rupture is only approximately 1% per aneurysm and year^2,3^. Nonetheless, neurosurgical and endovascular treatment options are also associated with relevant mortality and long-term disability risk^4^. The use of the different treatment options, therefore, remains controversial, and new criteria are sought to complement established aspects like patient age, aneurysm location, and its size^5,6^ in order to refine existing guidelines for treatment^1^.

Important information could, for instance, be contributed by hemodynamic and mechanical factors of the vasculature. Especially quantitative wall motion and pulsatile deformation data (caused by the cardiac cycle) is currently assumed to provide helpful predictive information on aneurysm rupture risk^1^. Despite well-described challenges of aneurysm wall motion imaging and image analysis such as interference of local and global motion patterns^7–10^, existing imaging studies indeed seem to support this assumption: As reviewed by Vanrossomme *et al*., successful detection and quantification of pulsatile-type aneurysm wall motion and correlation of wall motion to rupture status have already been reported^1^.

However, for standard imaging modalities like magnetic resonance imaging (MRI), computed tomography angiography (CTA), and 3D rotational angiography (3D-RA), measured image intensity is directly linked to (depending on the actual protocol) contrast agent inflow or changes of the blood velocity. Associated intensity fluctuations hinder image analysis and interpretation and might be mistaken as physical deformations; yet, related uncertainties are largely neglected. Further considering the typical spatial resolution of aforementioned imaging modalities, the potential misinterpretation of image intensity fluctuations becomes even more critical, since intracranial vessels and pathologies usually measure only a few millimeters in diameter, and expected wall motion and deformation magnitudes are even smaller and eventually below the spatial image resolution.

Our work aims at analyzing respective imaging and image analysis uncertainties regarding the quantification of pulsatile deformation of cerebral vessels and aneurysms. It was designed as a multi-imaging modality ground truth study: A physical flow phantom was developed, 3D-printed, and applied for MRI, CTA, and 3D-RA imaging. Phantom materials and structure dimensions were chosen to warrant ‘no motion’ scenarios (i. e. reliable ground truth data for subsequent image analysis) during physiologically plausible flow conditions. Precision and flexibility inherent to 3D printing allowed different cerebral structure geometries to be rapidly designed and projected effects to be studied in relation to geometry complexity. The (not existing) wall deformation in the ground truth image sequences was estimated and quantified by an established non-linear registration-based image analysis approach, and differences between the imaging modalities were analyzed. The present study thereby extends our related conference contribution^11,12^, which contained first preliminary data obtained by our experimental setup using MRI imaging sequences.

The remaining part of the manuscript is structured as follows: Further methodical aspects of the study like additional details on the flow phantom and the performed experiments and respective results are described in the Methods and Results sections. However, to allow the reader to put the data into context directly, the following section first refines the review in Vanrossomme *et al*.^1^ and gives an overview of related studies and their results concerning cerebral aneurysm wall motion quantification. The existing data is then taken up and discussed given the results of the present study in the Discussion section, leading to final remarks in the Conclusions section.

## Cerebral Aneurysm Wall Motion Quantification: Overview of Related Work

An association between wall motion and aneurysm rupture has already been suggested in the initial work of Meyer *et al*.^13^. Using phase-contrast MR angiography (PC MRA), the pulsation-related change in ruptured aneurysm volume was reported to be (51% ± 10%), compared to (17.6% ± 8.9%) for non-ruptured aneurysms. Aneurysm volume estimation relied on manual measurements of aneurysm diameters along *x*, *y* and *z* image axes and an assumed spherical aneurysm geometry. Given the simplicity of the method, plus additional uncertainties due to, e. g., potential flow artifacts, caution is required with respect to the interpretation of the results^1^. Nevertheless, Hayakawa *et al*. as well as Ishida *et al*. also observed aneurysm wall motion and pulsating blebs in 4D-CTA data^9,10,14,15^. During surgery, wall motion positions could even be confirmed to be aneurysm rupture sites for two patients^10^ and pulsating blebs as rupture points^9^. Moreover, aneurysm pulsation was more frequently observed for ruptured aneurysms^14^. These observations further substantiated the early results of Meyer *et al*. and the suggested link between wall motion and impending aneurysm rupture^13^, but they were only based on visual assessment of aneurysm wall motion. As a natural next step, numerous studies aimed at image-based quantification of pulsatile aneurysm wall motion. Following the review of Vanrossomme *et al*.^1^, an abridged overview is given in Table 1, enriched by respective information about image resolution and exploited wall motion quantification approaches that are in the focus of the present work. Most of the studies directly worked on *in vivo* data, with typical imaging modalities being aforementioned PC MRI/MRA, 4D-CTA, and 3D-RA. From a perspective of image analysis, two approaches dominate: threshold- and registration-based aneurysm dynamics and wall motion quantification. Thresholding mainly refers to separating vasculature and structures of interests from the image background. Window/level settings are usually operator-specifically chosen. The resulting images and segmented structures are then used to calculate changes in volume over time or the like^16–18^. Such methods are, however, observer-dependent (in the case of a manual selection of thresholds). Furthermore, intensity fluctuations due to changes in blood velocity or inflow of contrast agent are usually not explicitly accounted for and introduce additional uncertainties during quantification of aneurysm deformation and cardiac cycle-related wall motion.

**Table 1:** Previous studies on aneurysm wall motion (WM) detection/quantification in patient image data.

Authors	Image modality	Image resolution	WMO	WMQ	WM(Q) assessment
Meyer *et al*.^13^	PC-MRA	unclear	15/16	1.0–1.5 mm^a^	manual
Wardlaw *et al*.^27^	PD-US	unclear	yes	53%^b^	manual
Kato *et al*.^28^	4D-CTA	unclear	10/15	no	unclear
Hayakawa *et al*.^10^	4D-CTA	unclear	4/23	no	visual inspection
Ishida *et al*.^9^	4D-CTA	unclear	13/34	no	visual inspection
Dempere-Marco *et al*.^29^	3D-RA	unclear	2/3	yes	registration
Oubel *et al*.^19^	3D-RA	unclear	4/4	0.5 mm	registration
Oubel *et al*.^20^	3D-RA	0.07–0.28 mm	10/18	0.0–0.29 mm	registration
Karmonik *et al*.^16^	2D PC-MRI	0.625 mm	7/7	0.15 mm (range: 0.04–0.31 mm)^c^	semi-automatic, threshold-based
Hayakawa *et al*.^14^	4D-CTA	unclear	24/65	no	visual inspection
Zhang *et al*.^23^	3D RA	0.154 mm	1/2	yes	registration
Kuroda *et al*.^17^	4D-CTA	0.25–0.5 mm	yes	5.40% ± 4.17%^d^	threshold-based
Firouzian *et al*.^22^	4D-CTA	0.23 mm	19/19	0.17 ± 0.10 mm^e^	registration
Hayakawa *et al*.^15^	4D-CTA	0.5 mm	20/56	no	visual inspection
Illies *et al*.^18^	4D-CTA	0.39 mm	yes	yes	semi-automatic, threshold-based

The studies are listed in chronological order. Image resolution refers to the in-plane spatial resolution of the reconstructed data. WMO: wall motion observed; if numbers are given, they refer to the frequency of wall motion observation. WMQ, wall motion quantification. PC-MRA: phase-contrast MR angiography; CTA: CT angiography; 3D-RA: 3D rotational angiography; PD-US: power Doppler ultrasonography.

^a^Reported as typical change in size of ruptured aneurysms in at least one dimension.

^b^Average increase of aneurysm cross-sectional area between diastole and systole.

^c^Average wall displacement, evaluated in 2D slices.

^d^Cardiac cycle-related aneurysm volume changes.

^e^Aneurysm diameter change.

Registration-based cerebral aneurysm wall motion quantification has been initiated by Oubel *et al*.^19^. The idea was to apply non-linear registration between a pre-defined reference image (like the first acquired image frame) and the other images of the respective temporal image sequence. The resulting deformation fields are assumed to represent pulsatile deformation with respect to the reference time point. In particular, Oubel *et al*. applied deformation fields computed in high-frame-rate DSA (digital subtraction angiography) to automatically propagate landmarks that were manually located on the aneurysm wall as represented in the first DSA frame^19,20^. Aneurysm wall motion was then quantified by Euclidean distances between original and propagated landmark positions. A similar application of non-linear registration for quantification of cardiac cycle-related aneurysm dynamics has also been reported for 4D-CTA^21^. As the applied non-linear registration approaches are intensity-based, they are (depending on the imaging modality) sensitive to inhomogeneities of contrast distributions^20^, contrast inflow and/or changes of flow velocity, and image noise. Without appropriate quantification of such uncertainties, interpretation of computed deformation fields and derived quantities is, therefore, hardly feasible. Due to the absence of ground truth *in vivo* deformation data, quantification of related uncertainties during (semi-)automatic image analysis is usually based on phantom data. For instance, Firouzian *et al*. and Zhang *et al*. simulated image sequences and thereby estimated uncertainties of registration-based quantification of cardiac cycle-related aneurysm volume changes to be in the order of 4% and below 10%, respectively^22,23^. Such *in silico* phantoms, however, almost always simplify details of the imaging process and resulting effects (system noise, occurrence of artifacts, etc.). In this regard, physical phantoms (also referred to as *in vitro* phantoms^23^) add reliability. For instance, Kuroda *et al*. imaged a syringe filled with normal saline and determined obtained volume changes of 0.248% as an indicator of insignificant changes^17^. The influence of actual flow dynamics was, however, not considered. In turn, Yaghmai *et al*., Umeda *et al*. and Zhang *et al*. constructed physical (flow) phantoms that allowed for illustration of the feasibility of aneurysm wall motion imaging by means of 4D-CTA and 3D-RA^7,23,24^. Similar to aforementioned *in vivo* studies, exact aneurysm deformation data was again not known or reported for these phantoms; thus, feasibility was demonstrated qualitatively, but uncertainties regarding wall motion quantification remain. This shortcoming of previous studies represented the motivation of the present study.

## Methods

### Design and fabrication of flow phantom

**Figure 1:**
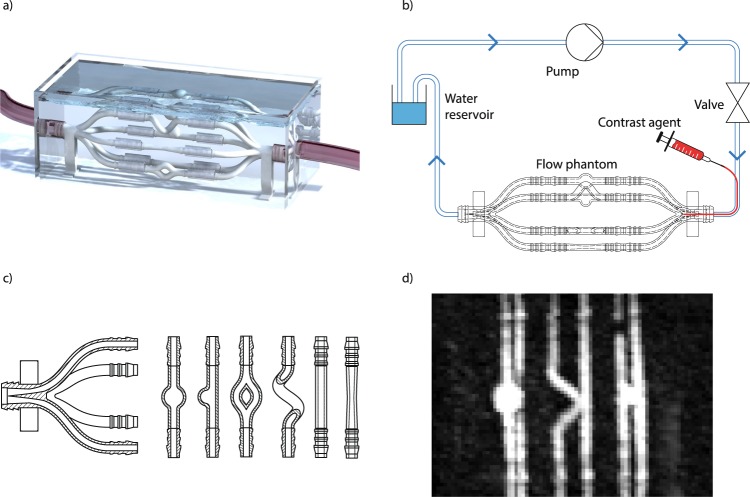
Flow phantom design and schematic representation of experimental setup. (**a**) Computer aided design, (**b**) measurement setup, (**c**) technical drawing of the phantom structures (flow distributor, two-sided aneurysm, one-sided aneurysm, bifurcation, helix, straight tube, stenosis), (**d**) maximum intensity projection of MR scan (TWIST), from left to right: one-sided aneurysm, straight tube, two-sided aneurysm & helix, bifurcation.

The physical flow phantom designed to provide ground truth data for assessment of pulsatile deformation quantification uncertainties is illustrated in Fig. 1(a). The phantom consists of three main parts: An inflow structure, modular models of the cerebral vasculature structures of interest, and the outflow (similar to the inflow structure, subsequently referred to as flow distributor). Technical drawings of the structures are shown in Fig. 1(c). The flow distributor features one intake and six smaller outflows that are connected to the vasculature-like structures. To eliminate unnecessary pressure changes in the vasculature structure models, the sum of the cross-sectional area of the outflows is designed to be identical to the cross-sectional area of the distributor intake. To avoid introducing turbulent flow, the fluid flowing into the distributor is further gradually divided into the six outflow tubes using a spike. For the present study, we designed six different vascular-like geometries to allow us to analyze imaging-related effects on automated flow-related deformation quantification depending on geometry complexity: straight tube, stenosis, helix, bifurcation, double-sided aneurysm, one-sided aneurysm. All structures were designed for three different inner diameters (${\varnothing }_{i,1}\,=\,4$ mm, ${\varnothing }_{i,2}\,=\,3$ mm, ${\varnothing }_{i,3}\,=\,2$ mm; wall thickness = 1 mm) of the inflowing tube to also study the diameter influence on the image analysis results. Technical drawings of the structures and a maximum intensity projection after MR imaging are shown in Fig. 1(c,d). The flow distributors and the vasculature-like structures were 3D-printed using only MRI-compatible materials (Verowhite, Polyjet). Relative to the applied pressure, the printing material has a high Young’s modulus, which ensured that the structures did not deform substantially during the experiments (cf. supplemental material S1). The general measurement setup is shown in Fig. 1(b). It consists of four parts: a pump, a water reservoir, a valve, and the physical flow phantom itself. The pump exerts a constant pressure on the closed liquid loop, generating a continuous velocity flow. The flow can be interrupted using the valve, triggered at the desired rate (here, e. g., 60 bpm) using a micro-controller to mimic the mechanic action of the heart and to obtain a physiologically plausible pulsatile flow profile.

### Imaging and dataset description

To ensure that all 3D-printed structures of the flow phantom (printing resolution: 0.04 mm) were accurately manufactured as designed, they were scanned using a HR-pQCT (high-resolution peripheral quantitative computed tomography) with a spatial resolution of 0.04 mm. Using this high-resolution imaging data, the geometries were analyzed before the actual study; defective structures were replaced. For imaging under flow conditions, three different imaging modalities were used: MRI was performed using a 3T scanner (Siemens Magnetom Skyra). To study effects of MR intensity variations due to contrast media inflow, contrast-enhanced TWIST (time-resolved angiography with interleaved stochastic trajectories; 4 ml GdDTPA-BMA [Omniscan], dilution 1:5) imaging was applied. 4D-Flow MRI was further utilized to analyze the influence of flow velocity change-induced intensity variation. CT angiography was performed using cine mode imaging of a Siemens SOMATOM Definition AS scanner and Imerson 400 (10 ml) as contrast agent. Finally, a Philips AlluraXper was used to acquire 3D-rotational angiography (3D-RA) data (contrast agent similar to CT imaging). Spatial image resolution after reconstruction was 1.3 mm and 1.0 mm for the MRI-TWIST and MRI-Flow sequences (isotropic resolution), 0.586 × 0.586 × 2 mm for CT, and 0.253 × 0.253 × 1 mm for 3D-RA imaging. All modalities and sequences were applied to imaging of all mentioned six different vasculature-like 3D-printed structures with the different inner diameters ${\varnothing }_{i,1}$ − ${\varnothing }_{i,3}$, resulting in 18 different spatiotemporal image datasets for each single imaging sequence. The datasets are available from the corresponding author on reasonable request.

### Automated quantification of pulsatile deformation

**Figure 2:**
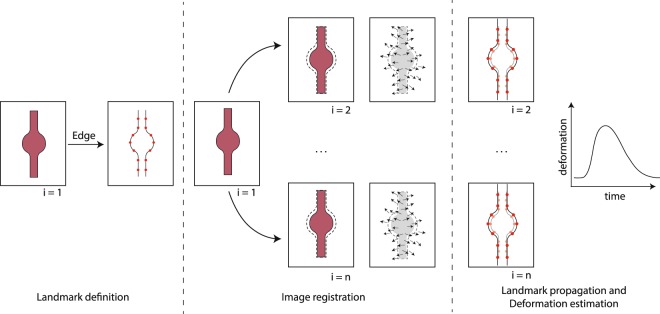
Deformation estimation approach: Using the edge information of the phantom structures, landmarks are automatically generated for image frame *i* = 1. Non-linear registration was used to compute deformation vector fields with respect to frame *i* = 1. The generated landmarks are then propagated using the computed vector fields, allowing for the estimation of wall deformation.

To avoid the observer dependency inherent to semi-automatic/threshold-based deformation quantification, the current study builds on the work of Oubel *et al*.^19,20^ and their registration-based approach to analyze wall deformation. The applied method is sketched in Fig. 2. Yet, different to Oubel *et al*.^19,20^, we automatically selected (pseudo-)landmarks. In a first step, such landmarks were generated on the borders of the structures as represented in the first frame of the acquired image sequence. To extract the structure borders, a Sobel edge filter was applied to the first image frame with the structures being visible (for discussion of the effect of the choice of the edge filter on the computed results see supplemental material S2). In order to analyze the several structures separately, a region of interest (ROI) was defined manually for each structure. In each of these ROIs, 150 random edge points were sampled, which were used as landmarks. In the second step, all frames of the image sequence were non-linearly registered to the first frame using the Elastix framework^25^. In detail, a multi-resolution free-form deformation (FFD) registration with cubic B-spline interpolation functions and mutual information as distance measure was applied. B-spline-based FFD registration has been discussed to be less sensitive to intensity variations due to, for instance, contrast media distribution inhomogeneities than common optical flow approaches^20^. The registration script, including applied registration parameters, is provided as supplemental material S3. The resulting deformation fields are then applied to propagate the landmarks over time. The computed landmark displacements were interpreted as a measure of estimated wall motion and compared against the ground truth, i. e. *no* deformation.

## Results

After ensuring proper fabrication of the phantom parts by HR-pQCT scanning and visual inspection, all measurements (Flow/TWIST MRI, 4D-CTA, 3D-RA) could be acquired as described. Flow MRI further allowed for assessment of during-experiment flow velocities. With approximately 80–100 cm/s, they were in the range of reported physiologically plausible flow velocities^26^ and met the assumptions underlying the structure deformation estimation detailed in supplemental material S1.

**Figure 3:**
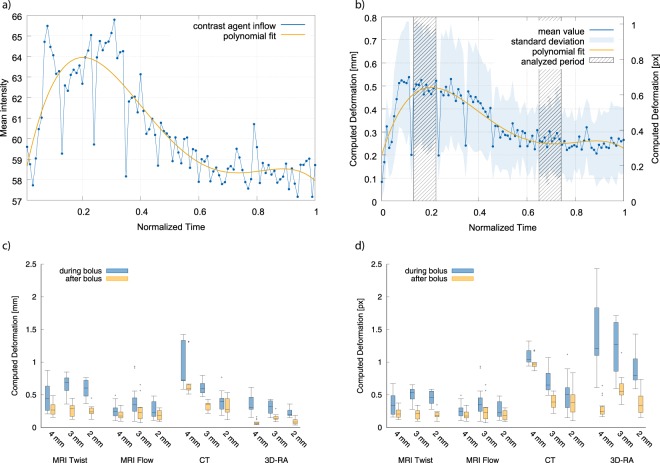
Inflow of contrast agent and results of landmark deformation estimation. (**a**) Inflow of contrast agent and resulting intensity in- and decrease of an exemplary MRI TWIST dataset, (**b**) estimated deformation of the phantom structures (average across all six structures), based on registration-based landmark deformation of exemplary MRI TWIST dataset, (**c**,**d**) overview of estimated deformation, given in mm (**c**) and px (**d**) for all imaging modalities (MRI [TWIST, Flow], CT, 3D-RA) during (blue) and after (yellow) contrast agent bolus.

Consequences of contrast agent inflow on image intensity are illustrated in Fig. 3(a). The figure shows the mean image intensity over time as observed in regions of interest that enclose the vasculature-like phantom structures (here: ${\varnothing }_{i}=4$ mm) as observed by TWIST MRI. A clear correlation between contrast agent inflow and image intensity is apparent, and the analysis of its influence on automated image-based estimation of vessel and aneurysm wall motion has been the motivation of this study. It should, however, be noted that respective intensity fluctuation not only represent potential obstacles for automated wall motion analysis but can also fool human observers (see the movie of a Flow MRI dataset provided as supplemental material). Thus, both the movie and Fig. 3(a) already strongly indicate difficulties with respect to analysis accuracy when (semi-)manual threshold-based vessel segmentation and wall motion quantification are applied. The influence of intensity variations on registration-based wall motion quantification is, however, less obvious. For illustration purposes, Fig. 3(b) shows the time-dependent registration-based estimated landmark displacement that corresponds to Fig. 3(a). Although using mutual information, i. e. a standard cost function for multi-modal registration that was selected to minimize the influence of intensity variations across time on registration outcome, and despite the fact of having imaged a static phantom and structure geometry, non-zero deformation is estimated. In addition, the picture not only shows a more or less constant offset that would indicate unintended adaptation of the registration algorithm to, e. g., random image noise; a clear correlation between the estimated deformation in Fig. 3(b) and the changes in intensity in Fig. 3(a) can be observed. Such data could easily be misinterpreted as cardiac cycle-related wall motion, especially since the deformation measures obtained in the period of bolus arrival are significantly higher than those after bolus decay (*t*(1199) = 69.96, *p* < 0.000001 for paired *t*-test of the periods highlighted in Fig. 3(b)).

Respective deformation measurements are summarized in Fig. 3(c,d) for all imaging modalities (TWIST MRI, Flow MRI, 4D-CTA, 3D-RA). It can be seen that, similar to TWIST MRI, non-zero deformation has been estimated for every single imaging modality (between 0 and approx. 1 mm). Differences are nevertheless apparent with respect to the estimated deformation magnitudes: Aforementioned effects were most prominent for TWIST MRI (0.36 mm) and 4D-CTA (0.55 mm), followed by 3D-RA (0.16 mm) and Flow MRI (0.24 mm). However, this is only part of the entire picture. The imaging modalities cover a wide range of spatial resolutions (in-plane resolution from 0.225 mm to 1.3 mm). To account for these differences, Fig. 3(d) provides deformation data as fraction of the in-plane pixel spacing. This reveals that the estimated deformation varies around one pixel for all contrast agent-based imaging modalities. Thus, contrast agent inflow can be seen to have a significant influence on automated (here: registration-based) quantification of cardiac cycle-related wall motion.

### Deformation estimation and structure complexity

**Figure 4:**
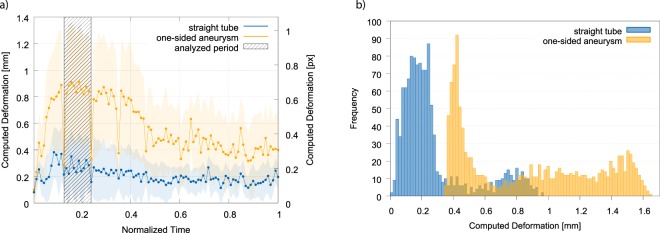
Differences in estimated deformation for flow phantom structures (imaging modality: MRI TWIST). (**a**) Estimation of wall deformation for two structures (straight tube, one-sided aneurysm), showing distinct deformation differences between the structures; (**b**) deformation histogram for the one-sided aneurysm and the straight tube as computed during the contrast agent inflow period.

Up to this point, deformation measures were averaged across all six structures. To further investigate the hypothesis that estimated deformation is not only dependent on the inflow of contrast agent, but also on the complexity of the geometry, a respective detailed analysis of the results has been performed. The data is summarized in Table 2. All used imaging modalities show a different extent of deformation for the different structures, indicating that the quantified deformation is dependent on the structure geometry. For further illustration, Fig. 4(a) shows a comparison of the estimated deformation over time with respect to the initial image frame for the 4D-TWIST data. In this case, the straight tube structure (${\varnothing }_{i}$ = 4 mm) and the one-sided aneurysm structure (${\varnothing }_{i}$ = 4 mm) are compared. While no correlation between estimated deformation and contrast agent inflow can be identified for the straight tube, such a relation is clearly visible for the more complex aneurysm structure. The potential for misinterpretation becomes even more evident if comparing the deformation data of the landmarks as observed for the two structures during bolus arrival (the highlighted period in Fig. 4(a)): As shown in Fig. 4(b), the data represents deformation distributions or histograms with only little overlap. Statistical testing using a *t*-test reported a significant difference between the straight tube and one-sided aneurysm during the inflow of contrast agent (*t*(899) = 29.19, *p* < 0.000001; evaluated frames are marked in Fig. 4(a)). From a clinical perspective, the data could be (mis-)interpreted as a hint that aneurysms exhibit stronger wall motion than straight vessels.

**Table 2:** Deformation estimation for all imaging modalities and structures (1 – straight tube, 2 – stenosis, 3 – bifurcation, 4 – helix, 5 – one-sided aneurysm, 6 – two-sided aneurysm).

Modality	Size	Mean (Bolus) [mm]						Std [mm]					
		1	2	3	4	5	6	1	2	3	4	5	6
TWIST	2 mm	0.41	0.67	0.67	0.63	0.60	0.59	0.06	0.10	0.10	0.21	0.03	0.17
	3 mm	0.61	0.67	0.75	0.72	0.80	0.40	0.04	0.13	0.04	0.04	0.07	0.04
	4 mm	0.27	0.22	0.63	0.37	0.85	0.52	0.04	0.01	0.02	0.01	0.03	0.02
Flow	2 mm	0.45	0.18	0.40	0.14	0.19	0.25	0.03	0.01	0.01	0.03	0.03	0.01
	3 mm	0.20	0.80	0.42	0.41	0.28	0.21	0.13	0.21	0.03	0.07	0.03	0.06
	4 mm	0.27	0.46	0.26	0.26	0.13	0.18	0.03	0.03	0.08	0.02	0.03	0.03
CT	2 mm	0.39	0.61	0.42	0.23	0.41	0.34	0.01	0.15	0.03	0.05	0.02	0.19
	3 mm	0.61	0.65	0.56	0.55	0.67	0.56	0.09	0.16	0.03	0.04	0.02	0.06
	4 mm	0.73	0.72	0.61	1.41	1.32	1.08	0.01	0.00	0.00	0.01	0.02	0.42
3D-RA	2 mm	0.24	0.20	0.24	0.22	0.26	0.20	0.07	0.01	0.10	0.06	0.07	0.02
	3 mm	0.31	0.33	0.33	0.25	0.35	0.26	0.12	0.10	0.14	0.09	0.12	0.10
	4 mm	0.32	0.37	0.35	0.35	0.32	0.41	0.03	0.19	0.16	0.13	0.07	0.23
**Modality**	**Size**	**Mean** (**After**-**Bolus**) [**mm**]						**Std** [**mm**]					
		**1**	**2**	**3**	**4**	**5**	**6**	**1**	**2**	**3**	**4**	**5**	**6**
TWIST	2 mm	0.30	0.21	0.30	0.21	0.22	0.28	0.00	0.10	0.13	0.07	0.02	0.05
	3 mm	0.31	0.29	0.37	0.21	0.29	0.15	0.05	0.12	0.07	0.08	0.10	0.02
	4 mm	0.21	0.19	0.35	0.20	0.46	0.26	0.05	0.03	0.03	0.06	0.05	0.04
Flow	2 mm	0.23	0.18	0.25	0.11	0.28	0.10	0.06	0.07	0.02	0.01	0.02	0.01
	3 mm	0.11	0.51	0.27	0.21	0.26	0.14	0.03	0.18	0.04	0.06	0.04	0.03
	4 mm	0.14	0.32	0.17	0.22	0.16	0.16	0.03	0.03	0.07	0.03	0.02	0.02
CT	2 mm	0.26	0.52	0.42	0.14	0.37	0.24	0.00	0.02	0.02	0.02	0.08	0.02
	3 mm	0.36	0.41	0.38	0.34	0.23	0.28	0.01	0.02	0.03	0.02	0.01	0.06
	4 mm	0.66	0.59	0.59	1.31	0.53	0.57	0.01	0.01	0.01	0.00	0.02	0.00
3D-RA	2 mm	0.10	0.09	0.10	0.10	0.08	0.11	0.08	0.05	0.07	0.02	0.02	0.05
	3 mm	0.13	0.21	0.14	0.14	0.15	0.13	0.04	0.07	0.05	0.01	0.03	0.01
	4 mm	0.08	0.06	0.06	0.07	0.09	0.08	0.05	0.01	0.01	0.01	0.06	0.04

For the other imaging modalities, similar relations were observed. Yet, the structure revealing the largest extent of deformation was not consistent across the imaging modalities. Nonetheless, the structures with the biggest expansion were typically the one-sided aneurysm, the stenosis or the bifurcation. Thus, the results indicate that for contrast agent-based imaging the complexity of the structure influences the estimated deformation.

## Discussion

The goal of the present study was to evaluate the influence of contrast agent inflow- and flow velocity variation-based changes in image intensity on (semi-)automated quantification of geometric deformation of cerebral vessels and pathologies, and especially aneurysms. To be able to operate on a reliable image dataset, a physical flow phantom was designed and 3D-printed that contained vasculature-like structures of different geometry complexity. The phantom allowed acquisition of ground truth, i. e. *no deformation* MRI, 4D-CTA, and 3D-RA image sequences. Adapting an established registration-based approach for wall motion quantification^19,20^, the study reveals that, e. g., intensity changes due to contrast agent inflow lead to computation of fictitious wall motion. Moreover, computed wall motion depends on the structure considered, with motion magnitudes increasing for more complex structures. These findings were consistent across the applied contrast agent-based imaging modalities.

From an application point of view, it has to be stressed that computed fictitious landmark motion (serving as a proxy for wall motion) magnitudes reside in the same order of magnitude than data previously reported for related *in vivo* studies, cf. Table 1. This suggests systematic overestimation of *in vivo*-detected wall motion when effects due to contrast agent inflow and image intensity changes are not corrected and accounted for. As details like parameter settings of the algorithms that have been applied for wall motion quantification are, however, often not described, no conclusive statement can be drawn at the moment. This, in turn, should be understood as a motivation to document technical approaches applied in the given context transparently.

In terms of potential limitations of our study, it can be argued that all measurements were performed using water instead of blood and that the different viscosity of the two fluids could lead to differences between *in vivo* and our *in vitro* measurements. However, the generated flow patterns have not been studied in detail and were not in the focus of the work; instead, only the effect of the inflowing contrast agent on the estimated deformation of the phantom structures was of interest. Therefore, the fluid in the flow circuit merely serves as a carrier medium for moving the contrast agent through our phantom structures. Further taking into account the typical spatial image resolution, we consider potential viscosity-related effects on the addressed deformation estimation to be small. In using water as main medium, we follow studies with a comparable study objective (e.g., Zhang *et al*.^23^, Umeda *et al*.^7^, Yaghmai *et al*.^24^).

Continuing with potential aspects infringing on the interpretability of our results, Fig. 3(a) reveals limitations regarding the pulse wave, which in our setup was controlled by a simple valve. While the simple valve generates a variation in flow velocity (providing plausible Flow-MRI data), the profile of the flow velocity is not engineered to precisely resemble a cardiac pulse wave. Since the valve closes completely, the fluid flow is interrupted, which leads to the drops in intensity visible in the figure. These intensity drops are not physiologically plausible; however, due to the separate registration of each individual image frame to the reference frame, they do not undermine the correlation between intensity variation and deformation estimation. On the contrary, since the drops in intensity can also be detected in the estimated deformation it reinforces the hypothesis.

In addition, the registration approach and parameters (here: implemented using Elastix^25^; motivated by Oubel *et al*.^20^) will also influence the computed deformation parameters. Imaging modality-specific adaptation of the registration settings could have reduced the fictitious wall motion magnitude. However, with such settings, the registration approach could have also failed to detect any existing wall motion using *in vivo* data. Thus, the exact relation between and differentiation of real deformation in *in vivo* data and the seeming deformation due to contrast agent inflow has not yet been investigated. This, in turn, could be studied using flow phantoms that exhibit reproducible deformation during pulsatile flow. We are currently working on the design of such phantoms; their design and the measurement setups to accurately measure in-experiment deformation of the structures are, however, complex tasks and beyond the scope of this paper.

Still, all remaining uncertainties do not counteract the observed computation of fictitious wall motion for the static geometry phantom. In contrary, they even underline the main conclusion of our study: Quantification of subtle effects such as pulsatile vessel and aneurysm deformation requires considering limitations of current imaging modalities and critical discussion thereof. From our perspective, maximum transparency concerning the applied image processing algorithms and parameters is necessary to render reported data reliable.

## Conclusions

Taken together, the experiments of our multi-imaging modality ground truth (i.e. static geometry) phantom study reveal that (semi-)automated quantification of cardiac cycle-related wall motion of cerebral vascular structures is subject to severe uncertainty. Specifically for TWIST MRI, 4D-CTA and 3D-RA, the uncertainty due to contrast agent inflow and related image intensity changes led to the computation of fictive vessel structure deformation over time. Such effects were even more prominent in more complex structures like aneurysms (compared to, e.g., straight tube-like vessels). In our opinion, it is further of particular importance that the computed fictitious deformation was of the same order of magnitude as (cerebral aneurysm) wall motion data reported in previous *in vivo* studies. This does not put respective data into question but highlights the importance of accompanying related publications by a detailed specification of utilized algorithms/parameters and, ideally, phantom ground truth experiments to illustrate appropriateness of the chosen technical approaches.

## Electronic supplementary material

Supplemental materials

Video.
