# Supplementary material for: Analysis of the influence of imaging-related uncertainties on cerebral aneurysm deformation quantification using a no-deformation physical flow phantom
Source: Sci Rep. 2018 Jul 20;8:11004. doi: 10.1038/s41598-018-29282-0 (PMC6054631; doi:10.1038/s41598-018-29282-0)
Supplement: Supplementary file 1 — Supplemental materials [file 41598_2018_29282_MOESM1_ESM.pdf]

Analysis of the influence of imaging-related uncertainties on cerebral aneurysm deformation quantification using a no-deformation physical flow phantom

Daniel Schetelig[1,*], Jan Sedlacik[2], Jens Fiehler[2], Andreas Frölich[2], Tobias Knopp[3,4], Thilo Sothmann[1,5], Jonathan Waschkewitz[5], and René Werner[1]

[1]University Medical Center Hamburg-Eppendorf, Department of Computational Neuroscience, Hamburg, 20246, Germany
[2]University Medical Center Hamburg-Eppendorf, Department of Diagnostic and Interventional Neuroradiology, Hamburg, 20246, Germany
[3]University Medical Center Hamburg-Eppendorf, Section for Biomedical Imaging, Hamburg, 20246, Germany
[4]Hamburg University of Technology, Institute for Biomedical Imaging, Hamburg, 20246, Germany
[5]University Medical Center Hamburg-Eppendorf, Department of Radiotherapy and Radiation Oncology, Hamburg, 20246, Germany
[*]d.schetelig@uke.de

Supplemental material S1

The estimation of the deformation of the flow phantom structures is conducted using Barlow's formula, which serves to calculate the tension (σ_t) due to internal pressure (p) in thin-walled structures (wall thickness: δ, radius: a) of a cylindrical pressure vessel:

$$\sigma_t = \frac{pa}{\delta} \tag{1}$$

Applying Hooke's law, using the Youngs modulus (E) and the Poisson's ratio (ν), the deformation ε_t can be described as

$$\varepsilon_t = \frac{pa}{E\delta}\left(1 - \frac{\nu}{2}\right). \tag{2}$$

This leads to a deformation of the cylinder wall

$$\Delta a = a\varepsilon = \frac{pa^2}{E\delta}\left(1 - \frac{\nu}{2}\right). \tag{3}$$

The material properties of the used filament are:

$E = 2000\ \mathrm{N/mm^2}$
$a = 3$ mm (i.e. outer diameter of 4 mm structure divided by two)
$\nu = 0.38$
$\delta = 1$ mm

The pump can provide a maximum pressure of $p = 0.035\ \mathrm{N/mm^2}$. To present a conservative estimate, the friction losses in the tubes are assumed to be zero. This assumption results in a deformation of

$$\Delta a = 1.28 \cdot 10^{-4}\ \mathrm{mm}. \tag{4}$$

Supplemental material S2

The generation of landmarks is a critical part of this study and is directly dependent on the generated edge images. We, therefore, tested multiple edge detection filters and compared the respective deformation results. In detail, we re-computed our results presented in the main manuscript for an exemplary dataset (TWIST, 4 mm two-sided aneurysm) using landmarks generated by Sobel filtering (this time using a different intensity threshold), Laplace filter, Prewitt filter, Kirsch filter, and Robinson filter. The results of the correspondingly estimated structure deformation are shown in the supplemental Fig. 1 that can be found below. In total, all edge detection filters provide similar results and are viable options to define the basis of automatic landmark definition. Statistical testing of the differences of the shown deformation distributions using a two-sample t-test reported no significant difference between the results of the various edge detection filters (for all distributions: all $t(98) < 0.27$, all $p > .78$).

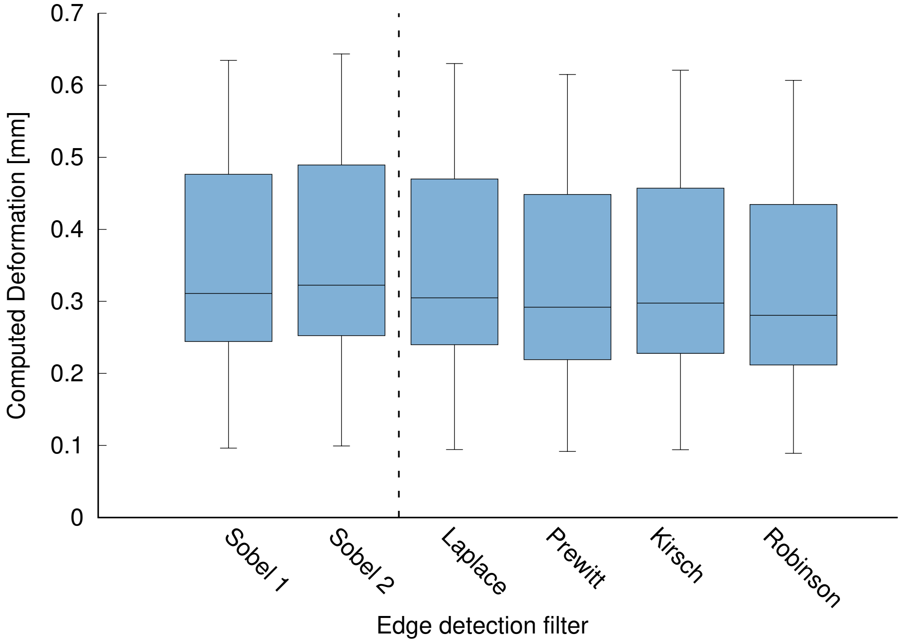

Figure 1. Effect of the choice of edge detection filters on deformation estimation. Investigated edge filters are Sobel filters with two different intensity thresholds, Laplace filter, Prewitt filter, Kirsch filter and Robinson filter.

Supplemental material S3

```
// BSplinePar

(FixedInternalImagePixelType "float")
(MovingInternalImagePixelType "float")

(FixedImageDimension 3)
(MovingImageDimension 3)

(UseDirectionCosines "true")


// **************** Main Components *************************

(Registration "MultiResolutionRegistration")
(Interpolator "BSplineInterpolator")
(ResampleInterpolator "FinalBSplineInterpolator")
(Resampler "DefaultResampler")

(FixedImagePyramid "FixedSmoothingImagePyramid")
(MovingImagePyramid "MovingSmoothingImagePyramid")

(Optimizer "AdaptiveStochasticGradientDescent")
(Transform "BSplineTransform")
(Metric "AdvancedMattesMutualInformation")
```

```
// ***************** Transformation ***************************

(FinalGridSpacingInVoxels 10 10 10)
(GridSpacingSchedule 6.0 6.0 6.0 4.0 4.0 4.0 2.5 2.5 2.5 1.0 1.0 1.0)

(HowToCombineTransforms "Compose")

// ******************* Similarity measure **********************

(NumberOfHistogramBins 32)

// ******************** Multiresolution ***********************

(NumberOfResolutions 4)

// ******************* Optimizer *****************************

(MaximumNumberOfIterations 200)

(MaximumStepLength 0.02)

// **************** Image sampling **********************

(NumberOfSpatialSamples 2048)

(NewSamplesEveryIteration "true")
(ImageSampler "RandomCoordinate")


// ************* Interpolation and Resampling ****************

(BSplineInterpolationOrder 1)
(FinalBSplineInterpolationOrder 3)

(DefaultPixelValue 0)

(WriteResultImage "true")

// The pixel type and format of the resulting deformed moving image
(ResultImagePixelType "float")
(ResultImageFormat "mhd")
```
