# Supplementary figures and images for: Analysis of the influence of imaging-related uncertainties on cerebral aneurysm deformation quantification using a no-deformation physical flow phantom
Source: Sci Rep. 2018 Jul 20;8:11004. doi: 10.1038/s41598-018-29282-0 (PMC6054631; doi:10.1038/s41598-018-29282-0)

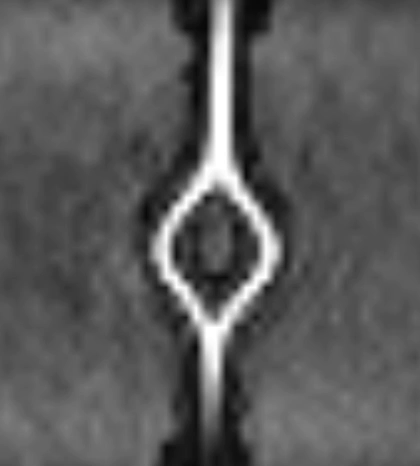

Supplement: Supplementary file 2 — Video. [file 41598_2018_29282_MOESM2_ESM.gif]
